# Exporting environmental democracy to international forums: Understanding the role of the Aarhus Convention

**DOI:** 10.1007/s13280-025-02293-8

**Published:** 2025-11-22

**Authors:** Nicola Sharman

**Affiliations:** University of Eastern Finland, Joensuu Campus, Yliopistokatu 2, P.O. Box 111, 80101 Joensuu, Finland

**Keywords:** Aarhus Convention, Environmental democracy, Procedural rights, Public participation, United Nations Framework Convention on Climate Change

## Abstract

This socio-legal paper examines how the duty under Article 3.7. of the Aarhus Convention to promote principles of environmental democracy in international environmental forums is being interpreted and operationalised in practice. A systematic content analysis of the parties’ 2021 and 2025 national implementation reports identifies uneven reporting and predominantly ad hoc approaches, focussing mainly on nationally based measures to facilitate the participation of states’ own publics, rather than collaborative initiatives to influence the design of participatory processes and outcomes of international institutions themselves. These findings point to a need for stronger institutional coordination, tailored forum-specific guidance, and more systematic monitoring and accountability mechanisms in order to strengthen Article 3.7’s operationalisation. More broadly, the paper also contributes to wider debates on the prospects of environmental democracy at scale, illustrating how the Aarhus Convention both exposes and tests the limits of efforts to democratise international and global environmental governance.

## Introduction

Applying ideals of environmental democracy to the international level has become a widely endorsed yet contested aspiration amid accelerating planetary crises. International institutions face pressure to pair the coordination of effective governmental responses with meaningful and equitable opportunities for civil society participation (Bäckstrand and Kuyper 2017). But while intergovernmental spaces traditionally reserved solely for states have increasingly opened to non-state actors and espouse values of democracy (Dingwerth et al 2020; Von Bernstorff 2021), the translation of democratic practices to forums beyond the nation state remains fraught with considerable theoretical and practical challenges (Bexell 2022). Existing efforts are typically criticised as fragmented, tokenistic, or ineffective (Sénit 2019; Mert 2019). Promoting environmental democracy at scale thus remains an open-ended project for its proponents, dependant on supportive institutional and legal frameworks, sustained state engagement, and scrutiny of evolving practice (De Búrca 2008; Pickering et al. 2020).

This paper examines the role of Article 3.7 of the 1998 Aarhus Convention as an underexplored legal mechanism designed to support the exportation of environmental democracy to the international level. The Convention, a landmark instrument at the intersection of international environmental law and human rights law, guarantees the three procedural rights of access to information, public participation and access to justice in environmental matters. Although its membership has remained limited largely to Europe and Central Asia, it is widely considered to have had a transformative impact in the domestic contexts of its member states (Sommermann 2017; Gellers and Jeffords 2018). But Article 3.7 reflects a vision to further extend the Convention’s reach to the international level by requiring parties to promote its principles in international environmental forums.

Despite the ambition of this provision, it has received little academic attention and remains poorly understood in practice. Early doctrinal interpretation concluded that the duty reflects ‘a normative investment in democratising international environmental decision-making’, but that its practical implications would require further assessment over time (Dannenmaier 2008). The most comprehensive study to date (Duyck 2015) examined the relationship between the Aarhus Convention and the UN Framework Convention on Climate Change (UNFCCC), showing how relevant stakeholders, the Aarhus Secretariat and the Aarhus parties have used institutional mechanisms to promote the Convention’s principles within the climate regime. Yet, the detail of this study is now dated and focuses on a single forum.

This paper revisits the question of how Article 3.7 is interpreted and operationalised through a socio-legal approach, by integrating earlier doctrinal assessments of the provision’s evolutionary and purpose-orientated character with systematic empirical analysis of reported state implementation. Drawing on the Aarhus parties’ 2021 and 2025 national implementation reports and complementary institutional documents, it identifies evolved interpretations, reporting patterns, practical limitations, and opportunities for strengthening the provision’s operational impact. The findings reveal uneven and predominantly ad hoc approaches, with a strong concentration on nationally based measures to facilitate the participation of states’ own publics rather than collaborative initiatives directed towards the participatory processes and outcomes of international institutions themselves. Using the UNFCCC as an illustrative case, the study also highlights how differing institutional features denote a need for updated and tailored guidance for key forums. More broadly, the paper situates these findings within the wider debate on the prospects of environmental democracy at scale, illustrating how the Aarhus Convention exposes and tests the limits of democratising international and global environmental governance.

## Theory and background

### Environmental democracy at international scale

Environmental democracy has evolved as a concept within political environmental scholarship over the last four decades, in tandem with the rise of environmental concerns on political agendas. According to its core definition, it contends that fulfilling normative ideals of democracy and achieving strong environmental outcomes are mutually reinforcing goals when supported by the right institutional conditions (Pickering et al. 2020). Proponents argue that robust democratic practice enables societies to navigate the complex and value-laden nature of environmental problems and fosters the sense of environmental citizenship that can sustain effective implementation of decisions. Sceptics, by contrast, consider democracy to be too cumbersome, short-termist and incapable of grasping technical and scientific complexity (Fischer 2018; Armeni and Lee 2021; Pickering et al. 2022).

These debates are increasingly salient amidst deepening environmental crisis and political polarisation. In Europe, prominent environmental activism and advocacy (Parks et al. 2023) sits alongside the spread of populist resistance to environmental regulation (Bosetti et al. 2025) and visible trends in democratic backsliding (Blauberger et al. 2025). But although there is compelling evidence of a positive relationship between democratic systems and environmental performance, the research remains inconclusive. Identifying and understanding the institutional conditions under which environmental democracy may be either supported or undermined therefore remains a pressing research agenda (Pickering et al. 2020, 2022).

As the need for global cooperation and solutions to planetary crises grows critical, the international dimensions of environmental democracy have become more pertinent. Democratic practices sit uneasily with traditional approaches to international law and governance, which rest on state consent and include only weak and uneven chains of democratic accountability (Bodansky 1999). But innovations in international environmental law to establish autonomous decision-making institutions and informal instruments, both designed to enable swift and adaptive decision-making, have eroded the role of state consent (Brunnée 2002; Palosaari 2025). These new governance approaches, politicisation of their subject matter, and proliferation of transnational actors have brought questions of democratic legitimacy deficits to the fore. A broad but contested literature in international political and democratic theory has developed using various lenses to examine how and to what extent such principles can be meaningfully realised at international scale (Bäckstrand 2006; De Búrca 2008; Stevenson and Dryzek 2014; Bexell 2022).

These factors have pushed international environmental institutions to espouse democratic principles of inclusivity, representation, deliberation, transparency and accountability and expand opportunities for civil society organisations to directly participate (Bäckstrand et al. 2017; Dingwerth et al. 2020; Dellmuth and Tallberg 2023; Sharman 2025). Yet participation remains fragmented and uneven, limited in depth and quality, unbalanced and vulnerable to co-option (Gereke and Brühl 2019; Mert 2019; UNFCCC 2022). Moreover, there is only limited evidence that current forms of non-state actor inclusion in international environmental institutions lead to improved environmental outcomes (Sénit 2019). Member states retain considerable influence over the design and implementation of participatory arrangements, meaning that the prospects of environmental democracy at scale hinge on the factors that sustain and drive states’ efforts to promote it’s realisation, including legal frameworks like the Aarhus Convention.

### The Aarhus Convention

The notion of environmental democracy first received international political endorsement in 1992 under Principle 10 of the Rio Declaration on Environment and Development, which declares that ‘[e]nvironmental issues are best handled with the participation of all concerned citizens.’ Building on this, the Aarhus Convention was adopted in 1998 under the auspices of the UN Economic Commission for Europe as a legal, rights-based codification of Principle 10 (Wates 2011; Barritt 2020). The Convention guarantees rights of access to information, public participation and access to justice (i.e. legal enforcement of the first two rights) in environmental matters and obliges the parties to maintain a clear, transparent and consistent framework to support them (arts. 1 & 3.1). These are broad concepts: public participation in particular has an inherently flexible scope for interpretation regarding the appropriate breadth and depth of engagement (Ebbesson 2021). But the Convention sets out a series of procedural standards to be applied to decision-making in domestic contexts relating to specific projects and activities, environmental policies and national regulations. Protection of these procedural environmental rights has come to be understood as an important legal mechanism supporting environmental democracy (Gellers and Jefford 2018; Pickering et al. 2020).

Reflecting ambition to extend its principles beyond its original membership base, the Aarhus Convention is open to accession by any country. But in practice, additional accessions have remained limited, and its 48 signatories remain located mostly in Europe and Central Asia. Preferring instead to pursue their own legal instrument to reflect regional conditions, 18 countries in the Latin American and Caribbean region have now ratified the 2021 Escazú Agreement, which mirrors the Aarhus rights while introducing other innovations such as explicit recognition of the substantive right to a clean and healthy environment, protections for environmental defenders and vulnerable groups, and strong commitments to capacity-building (Stec and Jendrośka 2019). For now, the Escazú parties are yet to establish a significant body of practice and other regions have not developed similar instruments.

Notably, the restricted regional membership base that the Aarhus Convention has retained also heightens the significance of the European Union’s membership, the member states of which make up the majority of Aarhus parties. First, the EU occupies a distinctive position as both subject and enforcer of the Convention: it requires its member states to transpose Aarhus obligations into national law,^1^ while EU institutions themselves must also comply with the Convention’s requirements.^2^ Second, the EU’s membership determines how its delegation engages in the Convention’s institutional processes. The EU delegation (comprising the Commission, the Council presidency, and Member States) agree a joint mandate in advance and speak with one voice at meetings in relation to general matters of development and enforcement of the treaty. Thus, the EU delegation is, as a collective, a dominant normative force (Berny 2025).

### Article 3.7

Article 3.7 reads: ‘Each Party shall promote the application of the principles of this Convention in international environmental decision-making processes and within the framework of international organisations in matters relating to the environment.’ The applicability of this provision has been interpreted widely (see Almaty Guidelines, para. 4) and can apply to forums engaging various kinds of actor (state or non-state), including negotiation of multilateral environmental treaties, institutional decision-making related to their subsequent implementation, or other intergovernmental bodies and international policy forums with environmental relevance or impact (hereinafter ‘international environmental forums (IEFs)’).

Article 3.7’s language reflects the challenges of translating the Convention’s objectives and provisions to international contexts, particularly given they are primarily designed for domestic application and in light of the Convention’s limited membership. The requirement only to *promote* principles recognises that different IEFs have various membership compositions, which might range from forums entirely comprised of Aarhus members to global IEFs where democratic values are not universally shared. Towards the latter end of the spectrum, Aarhus parties therefore can only attempt to exert influence through their roles as organisational members, participants and contributors (Dannenmaier 2008).

These constraints explain why Article 3.7 also does not refer to the procedural *rights* protected by the Convention, but rather its *principles*. Unlike rights, principles carry less normative weight or legally enforceable protections at individual level. It is likely that negotiators were seeking to avoid directly triggering the theoretical and practical questions of environmental democracy at scale by extending procedural rights to the international level. The final version also departs from a previous draft that would have required states to support the treaty’s *provisions*, a formulation that would have incorporated the concrete rules outlined in the text (Dannenmaier 2008). Beyerlin (2015) describes the Convention’s principles as its somewhat more abstract ‘substratum’. Though not explicitly defined, this can be understood to include all three pillars of access to information, public participation and access to justice as guiding values rather than rights, alongside cross-cutting principles such as non-discrimination, transparency, equity, and accountability. Dannenmaier (2008) characterised the resulting duty as one of ‘evangelism’ to ‘spread the good word’ of the Convention. Therefore, the formulation of Article 3.7 can be read as aiming to strike a balance between encouraging ambition while remaining realistic. But its lack of clarity and precision limit the provision’s legal effect. This continues to be reinforced by the fact that Article 3.7 has never been the subject of a compliance case brought to the Aarhus Convention’s Compliance Committee (ACCC).

Although the focus of this paper is the Aarhus Convention, it should be noted that the 2021 Escazú Agreement has a similar provision (art. 7.12). Its formulation is weaker, given that it does not require promotion of the Agreement’s broader principles but only *public participation* and contains an extra qualification that this only applies ‘where appropriate’ and ‘in accordance with the procedural rules on participation in each forum’. Given the early stage of the Escazú regime, little information is available that could give deeper insight into how this obligation is being interpreted. But, as will be discussed, it is relevant to this study in so far as it opens scope for collaborative action amongst the parties of the two regimes at international level in implementation of parallel obligations.

### The Almaty Guidelines

The Meeting of the Parties (MOP) is the Convention’s plenary body, convening roughly every three to four years. At its first meeting in 2002, states decided that guidelines on Article 3.7 should be developed (MOP-1, para. 31). The Almaty Guidelines were produced by a drafting committee and adopted at the next meeting in 2005 (MOP-2). Their stated purpose is to provide general guidance to the Aarhus parties in the context of the development, modification and application of relevant institutional rules and practices, as well as treatment of substantive issues within those forums. But the Guidelines also invite other interested states, NGOs and IEFs to use them as a source of inspiration (preamble 2, 3 & 4, paras. 1–3).

A series of ‘general considerations’ are set out, including the need to ensure international access is meaningful and equitable, open to the public at large, includes special measures for those with different capacity and circumstances, minimises undue influence, and is non-discriminatory based on citizenship or nationality (paras. 11–18). They also lay out more specific recommendations under the three Aarhus pillars. In relation to access to information, information should be provided in a clear, timely and proactive manner, in accessible and meaningful formats, by appropriate technical means, specific requests should be responded to as soon as possible, and any grounds for refusal should be interpreted restrictively (paras. 19–27). In relation to public participation, the Guidelines dictate that this should be equitable and as broad as possible. It will be enhanced through representation of diverse constituencies, which may include members of the public likely to be most directly affected, public-interest organisations, or others capable of contributing to or alleviating the problems under discussion. The Guidelines also suggest that ensuring participation is meaningful entails it being consultative in nature, initiated early enough to influence outcomes, supported by relevant documentation, and facilitated at all relevant stages unless clearly justified. Practical challenges of scale and the need for transparent and fair selection criteria are also acknowledged, while encouraging innovative and cost-efficient approaches (paras. 28–39). The access to justice element contains only a single dedicated paragraph encouraging the facilitation of public access to review procedures (para. 40).

Two limitations of the Guidelines are apparent. First, they primarily describe standards according to which IEF processes and procedures should be designed, rather than specifying what concrete steps the Aarhus parties themselves, as subjects of the Convention, should take to promote Aarhus-aligned procedural design. Second, the focus on the processes and procedures of IEFs themselves overlooks other dimensions of Article 3.7 that have subsequently been recognised, as discussed below.

### Post-2005 developments

From 2005 to 2011, Article 3.7 implementation efforts were led by a dedicated Task Force, which consulted with relevant IEFs, considered how the parties should report on their relevant activities, organised workshops, and prepared a report of good practices (Duyck 2015). On the basis of this work, in 2011 the MOP adopted a decision that marked a shift beyond the Almaty Guidelines’ focus on the procedures and processes of IEFs themselves (MOP-4a, paras. 9(b)-(c)). First, it called upon the parties to ‘provide access to information and enable public participation at the national level regarding international forums’. This clarifies the importance of states facilitating the engagement of their own publics in their contributions to IEFs, which the parties have full control over. Second, the MOP called upon the parties to promote the principles of the Convention ‘in the work programmes, projects, decisions, instruments and other substantive outputs of those forums’. This addition is more ‘outward looking’ towards the ways in which substantive activities emerging from the IEF, which may be delivered by various actors in either other national or transnational contexts, are implemented. The parties will only have limited influence over such outcomes. These new elements were also reflected in an amendment to the template for the member states’ national implementation reports, which the parties are required to submit every three to four years, via a series of sub-questions on Article 3.7 implementation (MOP-4a).

Since 2011, work on Article 3.7 has collectively continued under the ambit of the Working Group of the Parties (WGP), which oversees the implementation of the Convention’s work programme. Each of its annual meetings includes a thematic session on the topic of promoting the Convention’s principles in IEFs. These sessions focus on pre-agreed topical themes (e.g. specific forums, COVID-19 measures, the role of private corporate actors) and have been used for knowledge exchange, sharing of best practices and to call for particular actions from parties (Duyck 2015). The WGP is also mandated to convene workshops, consult experts, and provide meeting reports and recommended decisions to each MOP. In 2021, the parties mandated the WGP to focus on IEFs dealing with global environmental challenges, including threats to the marine environment, loss of biodiversity, and climate change (MOP-7, para. 10b).

## Materials and methods

### Treaty interpretation: a purposive approach

This paper’s methodological approach adheres to a purposive interpretation of the Aarhus Convention. Article 31(1) of the Vienna Convention on the Law of Treaties (VCLT) provides that a treaty shall be interpreted ‘in good faith in accordance with the ordinary meaning to be given to the terms of the treaty in their context and in the light of its object and purpose’. The VCLT further provides that any subsequent agreement, practice or relevant rules of international law may be considered relevant context and where the meaning of a treaty is ambiguous or obscure, supplementary means of interpretation may also be used (arts. 31.2 and 32). An interpretive approach that emphasises purpose thus allows the meaning of a treaty to expand and evolve to new surrounding conditions and is often employed by courts in the case of ambiguous texts or human rights instruments which have a value-based and ‘living’ character (Dothan 2019; Barritt 2020). At the same time, state practice provides a concrete and persuasive indication of how purpose is translated into action (Gardiner 2015).

In the case of the Aarhus Convention, the promotion of environmental democracy can be pinpointed as a clear and central purpose that should guide interpretation. Emily Barritt’s (2020) analysis affirms this overarching purpose by relying on a combination of formal sources such as the Convention’s negotiation record, Implementation Guide and Strategic Plans, as well as academic and political commentaries. Given the ambiguity of Article 3.7, this purpose serves as a valuable anchor according to which the provision’s more particular meaning and implications may be construed in an evolutionary way (Dannenmaier 2008). It is within this context that the parties elaborated on additional components of the Article 3.7 duty that the Almaty Guidelines did not originally address. It is also from this perspective that the national implementation reports become an important body of evidence: they provide insight into how states themselves, as both the primary legal subjects of the Convention and the key architects of its evolving practice, interpret Article 3.7 in light of this purpose.

### Content analysis of national implementation reports

The paper is based on a systematic content analysis of 80 national implementation reports (NIRs) submitted by the Aarhus parties during the two most recent reporting cycles in 2021 and 2025 (the sixth and seventh cycles, respectively).^3^ 46 out of 47 parties submitted a report in the 2021 cycle (the Netherlands being the exception) and, at the time of writing, 34 out of 48 parties have submitted a report in the 2025 cycle.^4^ The analysis was limited to these two most recent reporting cycles on the basis that the volume of information in successive rounds has visibly grown over time, with states encouraged to use their previous report as a starting point and add new information where relevant. The Aarhus Secretariat has also noted improvements in reporting on Article 3.7 implementation compared to previous years (Aarhus Secretariat 2021, para. 78). Restricting the analysis to these cycles therefore provides a snapshot of the most developed practice. While the incomplete submissions in 2025 introduces some asymmetry, their inclusion nevertheless ensures coverage of the most up-to-date reporting while retaining possibility for comparison across the completed 2021 reporting cycle.

Information relating specifically to Article 3.7 was manually identified and extracted from the NIRs, reports not submitted in English were translated using DeepL, and the data were uploaded to ATLAS.ti. A descriptive coding exercise was then carried out in three rounds across three themes: (a) implementation measures, (b) challenges of implementation, and (c) other information. For the first round, the reporting template’s sub-questions functioned as a reference point, before sub-categories were developed (Schebesta 2018; Saldaňa 2021; Naeem et al. 2023). A coding manual was maintained throughout the process to enhance consistency and reliability in coding decisions (Brook 2022). Finally, the data were analysed to identify qualitative patterns across the reports, while frequencies were also counted in order to generate some basic quantitative insights.

In order to contextualise the NIR analysis, other institutional documents which form part of the Convention’s normative framework were also consulted. These include MOP decisions, the Almaty Guidelines, reports of the WGP, and the results of a survey related to Article 3.7 implementation conducted by the Secretariat in 2023 (WGP 2023).^5^

### Limitations

Given the self-reported nature of the NIRs, they ultimately only offer selective information from the state’s point of view (Osae et al. 2024). Official guidance dictates that the reports should be prepared on the basis of consultations with the government, stakeholders and the public (ACCC 2007). But the extent to which each party has followed these guidelines and reflected those consultations in their report, particularly with regard to information on Article 3.7, is not verified. Moreover, it is likely that different practices have been adopted in terms of which officials and ministries are responsible for providing input to and drafting the reports. Any omissions do not necessarily confirm that relevant measures have not been taken, particularly given maximum length limitations. A further challenge that may contribute to underreporting is that measures to promote environmental democracy often operate across different levels of governance, yet states may not present them as relevant to Article 3.7. Lack of consistency and comprehensiveness among the reports, as discussed in the findings, would support the assumption that inconsistent practices in their drafting are being applied, thus limiting their comparability. Despite these limitations, the NIRs remain valuable as a window into state interpretation and practice.

## RESULTS

### General observations

The findings confirm that reporting on Article 3.7 implementation is uneven and often incomplete across the Aarhus parties, with no clear correlation to the parties’ geopolitical or developmental characteristics. While some reports were relatively rich in detail, many others contained significant vagueness, irrelevance, outdatedness, or selectivity, leaving the overall picture uncomprehensive and fragmented, and cross-country comparison difficult. Across both reporting cycles, thirteen reports provided either no information or only brief confirmations of compliance,^6^ and only six parties followed the template’s sub-questions for Article 3.7.^7^ Many reported activities were deemed to bear weak or no relevance to Article 3.7, for example measures taken to promote public involvement in domestic activities and policymaking, state participation in sharing domestic best practices, or descriptions of NGO activities without any clear indication of how they were supported by the state. Measures to inform or train relevant officials on Article 3.7 were also commonly reported (30 reports) but provided no insight into whether or how the training translated into practice.

The analysis also reveals that an ad hoc approach to implementation prevails. Despite agreed MOP decisions encouraging the development of action plans to systematically promote the Convention’s principles in all IEFs (MOP-6, para. 4(f); MOP-7, para. 6(f)), no parties report the existence of any such plan. Few report any legislation or policy that supports Article 3.7 implementation, while five parties specifically note its unsystematic nature.^8^ Even amongst more comprehensive reports, selective reporting in terms of types of measures and the specific IEFs they have been applied to was evident. Four parties highlighted that opportunities are limited by the differing rules, characteristics and decision-making structures of IEFs.^9^

Finally, several parties noted that they implement Article 3.7 in their capacity as members of the EU.^10^ Yet, the EU’s own reports lack any substantive detail on how Aarhus principles are adopted into its own institutional practices or how it promotes these principles in other IEFs, instead simply stating that ‘[i]n negotiations under multilateral environmental agreements, Commission representatives strive as far as possible to enable the participation of a wide circle of interested parties.’

### Substantive implementation measures

**Fig. 1:**
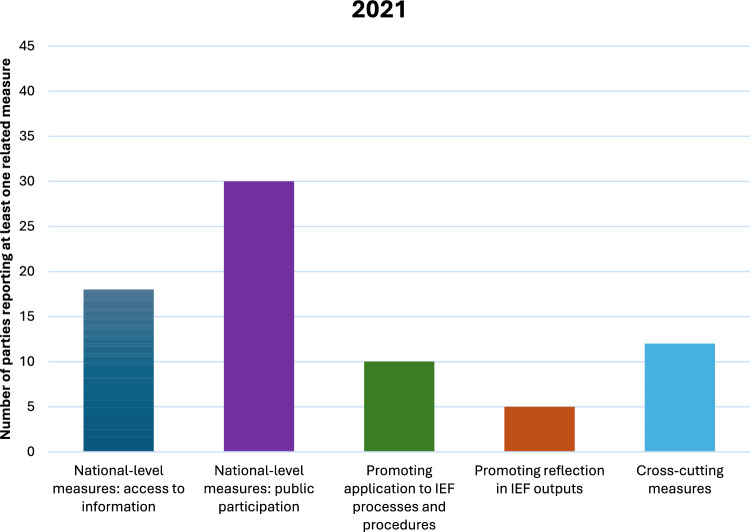
Frequency of  Article 3.7 implementation measures reported in 2021 National Implementation Reports

**Fig. 2:**
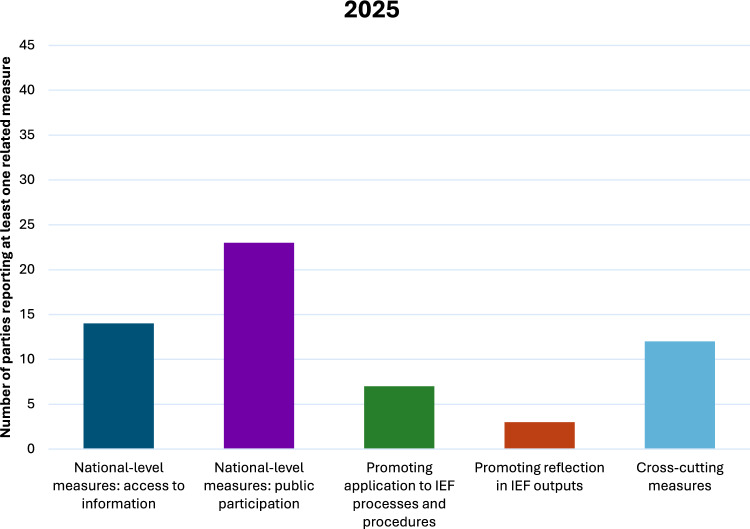
Frequency of Article 3.7 implementation measures reported in 2025 National Implementation Reports

Measures relating to each sub-question in the reporting template, as well as several cross-cutting measures, were identified throughout the reports, though with wide variation in detail and frequency. Figures 1 and 2 show the number of parties reporting at least one measure falling in each category in 2021 and 2025. The data demonstrate a clear imbalance in the reporting frequency of national-level measures compared to those taken to promote the Convention’s principles in the processes or outputs of IEFs themselves. This quantification does not, however, capture differences in the level of detail provided, nor consider the blurred boundaries and intersections that often arise in practice between access to information and public participation. Notably, no parties reported any measures relating to access to justice.

- Providing access to information on IEFs at national level

Efforts were identified to fall into two interrelated and often overlapping sub-categories. The first relates to facilitating access to information on international environmental issues and the activities of relevant IEFs in general. The second goes a step further, focussing on information about the state’s own role and contributions within an IEF. In both cases, reported methods of dissemination included hosting public and stakeholder information events, giving press briefings, or publishing meeting summaries, negotiation positions and delegation reports through official websites or digital media in local languages. These activities reflect recognition that states have a role to play in disseminating, translating, and contextualising information on IEFs for national stakeholders to enhance accessibility, understanding and engagement. However, the reports generally lacked sufficient detail to build a reliable picture of whether practices aligned with the Almaty Guidelines. Austria notably raised the issue of the need to balance transparency with confidentiality, which the Guidelines do not address. Others also described tailoring communication to different audiences, using more proactive and close engagement with expert or trusted national NGOs and relying on them for wider dissemination.

(b) Facilitating public participation in national contributions to IEFs

This category could be further broken down into three channels. The reporting template suggests two dimensions: inviting NGO members to participate in national delegations to IEF meetings and involving NGOs in forming official national negotiation positions. These two measures were widely reported. A third option is to involve the public in the preparation of other national submissions to IEFs, such as implementation reports. Descriptions of how national stakeholders were able to participate in the latter two varied widely, ranging from public consultations, preparatory working groups, public councils, bilateral ministerial meetings, or including civil society representatives on government advisory bodies. Several parties notably also referred to facilitating public participation in preparing national positions on EU matters^11^ or in processes relating to the environmental dimensions of ongoing EU membership negotiations.^12^ The NIRs were typically, again, very light in the level of detail given on concrete practices or standards applied. For example, no party explained their selection process for delegation members or what such inclusion entitles them to do exactly. However, a number of parties do report specifically engaging directly affected constituencies, in most cases youth, but also indigenous communities, women and people with disabilities.^13^

(c) Promoting application of Aarhus principles to IEF processes and procedures

Despite being the core focus of the Almaty Guidelines, reporting on this element was both infrequent and usually vague. Several reports nonetheless offer partial insights into potential avenues for implementation in practice. Beyond making formal statements or negotiation positions in institutional settings, which only a small number of reports allude to, others highlight softer or indirect approaches. For example, France reports sponsoring an NGO initiative to promote the Guidelines to delegates on the sidelines of two UNFCCC summits, Finland has endorsed a voluntary political pledge to support stakeholder participation in the High-Level Political Forum to the UN, and five countries report engagement in side events connected to the main IEF process.^14^ Five countries also reported initiatives to engage the public while presiding over or hosting major IEF meetings, thus recognising that holding such a position puts parties in a unique position of power and responsibility to enhance opportunities for public participation in IEFs in line with Aarhus principles.^15^

(d) Promoting reflection of Aarhus principles in substantive IEF activities and outputs

This dimension received overwhelmingly the least attention, with only five parties referencing specific submissions or contributions to IEF outputs that reflect Aarhus principles, either through formal statements, proposals or drafting input.^16^

(e) Cross-cutting measures

National capacity-building: Eleven parties reported capacity-building actions aimed at national NGOs, either by supporting them to participate in different types of international networks concerning environmental issues or to attend IEF meetings, in many cases by providing financial assistance, practical information, or ensuring badge access.^17^ Three also reported efforts to raise awareness of the Almaty Guidelines among NGOs and the wider public.^18^

Supporting wider adoption of procedural environmental rights: Only two parties reported supporting the broader recognition of procedural environmental rights beyond Aarhus states. Italy in particular emphasised that such broader uptake will help reduce resistance to promotion of relevant principles in IEFs and reported funding a project that contributed to development of the Escazú Agreement. Greece also reported participating in an initiative of Mediterranean countries aimed at encouraging accession to the Aarhus Convention itself.

Collaborative activities and technical assistance: Parties are encouraged to collaborate and provide technical assistance to host countries of major IEF conferences through several MOP decisions (MOP-4, para. 11; MOP-5, para. 5; MOP-6, para. 4–7 and 11(b)). However, there was almost no reporting of such activities, with only Austria making an outdated reference to its participation in a session of the 2010 Task Force.

## Discussion

### The strong focus on national measures

Although the findings provide evidence of state practice covering all dimensions of Article 3.7 implementation, they reveal a continuation of Duyck’s (2015) observation of a markedly stronger emphasis on national level measures and national capacity-building compared to promotion of Aarhus principles in the procedures or outputs of IEFs themselves. This emphasis can be attributed to both political and practical factors. Domestic initiatives are reinforced by stronger political incentives and public demand, while they also concern processes fully within governmental control. They are also simpler to document and justify in official reporting, while interstate negotiations on IEF processes and outputs often unfold behind closed doors, within negotiation blocs (as discussed below), or are couched within broader diplomatic agendas.

Yet this imbalance carries important risks connected to the regional and Europe-dominated membership of the Convention. National-level measures primarily benefit publics and NGOs within Aarhus jurisdictions, meaning that voices from non-Aarhus countries may not receive comparable support or opportunities. The effect could be to inadvertently reinforce representational imbalances in IEFs, contrary to the Convention’s democratic ethos. The existence of parallel agreements, particularly the Escazú Agreement, does mitigate this to some extent, but the fragmentation of coverage underscores the absence of a truly global guarantee of environmental democracy.

More fundamentally, these results resonate with sceptical perspectives on the prospects of scaling environmental democracy to the international level (e.g. Song 2012). While Article 3.7. creates an important normative mandate, institutional realities channel participatory practices towards the national sphere where states can act unilaterally. National measures thus risk substituting rather complementing implementation of other dimensions of the duty. Clear and explicit distinctions between these different dimensions is thus essential to avoid obscuring or excusing weaker collective efforts to push for participatory values to be integrated into IEF processes and outcomes.

At the same time, this paper’s overview underscores the need to look beyond institutional self-reporting and conduct deeper study of specific IEFs and countries. Honkonen (2023), for example, has conducted an empirical study of Finland’s national-level practices to facilitate the Finnish public’s participation in the UNFCCC on the basis of interviews with government officials and national stakeholders. Ethnographic studies of negotiations, and particularly internal EU processes, could help build understanding of efforts in the other dimensions.

### The limits of individual state action

The limited influence that can be exerted by any single Aarhus party over an IEF’s procedures and outputs underlines the importance of transparent collaborative efforts to promote the Convention’s principles. Joint identification of priority issues, common positions, and exchange of best practices can strengthen collective influence. The WGP thematic sessions provide a critical forum for such exchange. But the absence of their mention in the NIRs indicates a weak link between these sessions and any concrete action taken, while review of WGP decisions shows that clear, precise and action-orientated directions are uncommon. However, some examples show that this is not unfeasible, for example the WGP’s 2024 decision called on parties to promote Aarhus principles specifically during the next institutional review of the International Seabed Authority (WGP 2024, item 5viii). Decisions like these would help to operationalise Article 3.7, while future research could monitor participation and follow-up. Building regional cooperation with the parties to the Escazú Agreement, as underlined during the 2021 MOP (MOP-7, para. 5), will also be a key component of furthering this agenda.

The limited influence of individual states also spotlights the value of supporting the Aarhus Secretariat in Article 3.7-related work, which is mandated to collect and disseminate good practices via the Aarhus Clearinghouse online database, provide expert assistance to Aarhus parties and IEFs, and conduct other outreach activities such as training and workshops (MOP-4, para. 12; MOP-5, para. 7; MOP-6, para. 7; MOP-7, para. 11). Persistent funding shortfalls that may be undermining this work should be remedied (WGP 2024, item 5viii).

Finally, the reports indicate that bloc negotiation within IEFs also constrains individual state action. This is particularly evident in the case of the EU, given its influential position and the fact that Aarhus principles must be incorporated into the collective negotiation mandate. While there is normative power in advocating for Aarhus principles as a bloc, a significant lack of transparency or formal role for civil society in the mandate formulation process means that it could also be used as a political shield by individual Aarhus parties reluctant to promote the Convention’s principles in IEF processes and outputs (Honkonen 2025). While strategic confidentiality in international negotiations may justify keeping certain aspects of the mandate formation beyond public scrutiny, this dynamic nevertheless underscores the need for more comprehensive and transparent reporting on how agreed mandates reflect Article 3.7.

### The need for IEF-specific guidance

Several reports stressed that each IEF has distinct characteristics, including procedural rules, decision-making structures and established norms, underlining the need for tailored approaches and training for negotiators and ministers. Yet, in 2020 the Chair of the WGP noted that negotiators still had little awareness of the Aarhus obligations and that adapted guidance could promote good practice (WGP 2020, para. 67). In 2021, the MOP therefore tasked the Aarhus Secretariat with overseeing development of such materials (MOP-7, para. 11c). The findings of this study support that initiative, which could help bridge gaps between institutional discussion and practical implementation, as well as turning the somewhat abstract Article 3.7 commitments and outdated Almaty Guidelines into more meaningful and accountable standards.

The UNFCCC offers a clear example of why targeted guidance is needed. It was the most frequently mentioned IEF in the NIRs (32 reports), is a recurring theme at WGP sessions, and presents distinctive participation challenges. Established in 1992 under the UNFCCC and now principally governed by the 2015 Paris Agreement, the regime largely revolves around bottom-up procedural mechanisms to elicit increasingly ambitious national mitigation pledges and commitments related to adaptation, finance, technology transfer and loss and damage (Daniel et al. 2017). The regime is operationalised through an institutional structure headed by an annually-meeting Conference of the Parties (COP) and numerous subsidiary bodies.

Several features reinforce the need for tailored guidance. First, exceptionally high demand for observer accreditations puts great pressure on institutional resources and the ability to ensure fair access and representation (Yang and Walker 2025). Second, the institutional and procedural complexity of the Paris Agreement make the process extremely difficult to navigate and communicate to the public (CCES 2025). Third, the Paris Agreement institutionalises the role of non-state actors as implementors of climate action through several mechanisms, reflecting an ‘all hands on deck’ philosophy but intensifying risks of conflict of interest and greenwashing (Marquardt et al. 2022). Fourth, COPs feature a vast programme of side events and informal gatherings, the value of which is contested between either enriching the process with diverse voices or simply creating distracting noise (Xie et al. 2025). These contextual factors have a critical bearing on how Aarhus principles may resonate within the UNFCCC framework.

When it comes to national level measures, guidance could outline best practices for providing access to information and supporting public participation in key UNFCCC national submission processes, such as nationally determined contributions (NDCs), national adaptation plans, and inputs to the Global Stocktake. NDCs in particular are a central element of the regime as both expressions of national ambition but also as negotiation positions on allocation of responsibility (Leinaweaver and Thomson 2021).

In relation to the promotion of Aarhus principles within the UNFCCC process itself, guidance could also address the inconsistent procedural regulations found across different constituted bodies (Martinez-Blanco 2021). It could also highlight the standing agenda item on Arrangements for Intergovernmental Meetings (AIM), which is the primary formal avenue for decision-making on the COP process itself. Recent AIM decisions have aimed to increase procedural efficiency and enhance observer engagement through a series of recommendations and requests issued to parties, meeting chairs, the UNFCCC secretariat and incoming presidencies (UNFCCC 2024, para. 30–37). Guidance could address how the principles should apply to key topics which typically arise in these discussions, such as conflicts of interest, representation deficits, and informal participation spaces.

With regards to promotion of Aarhus principles in substantive UNFCCC activities and outputs, guidance could encourage the mainstreaming of Aarhus principles across all institutional outputs while also addressing particular domains of climate action where public participation is particularly important. For instance, the rules for carbon markets, offsetting projects, geoengineering, energy policy and access to independent grievance mechanisms are all topics which have been highlighted at recent WGP meetings.

Guidance should also highlight tracks of the regime which bear special resonance with Aarhus principles and in which the parties should therefore actively support strong outcomes. Most importantly, Article 6 of the UNFCCC obliges the parties to promote access to information, public participation, awareness, education and training, which is orchestrated through the Action for Climate Empowerment work program (Lika 2025). Also relevant is the Gender Action Plan, which seeks to ensure gender-responsive climate action including women’s participation, and the Local Communities and Indigenous Peoples’ Platform which aims to integrate traditional, indigenous, and local knowledge systems into the UNFCCC process (UNFCCC 2015, para. 35).

## Conclusion

This study strengthens understanding of the duty under Article 3.7 of the Aarhus Convention to promote its principles in international environmental forums by examining how states report implementation. The findings build on evolved interpretations of the duty since the adoption of the Almaty Guidelines in 2005, now encompassing national-level measures, measures to promote Aarhus-aligned design of IEF processes, and efforts to integrate Aarhus principles into IEF outputs. Yet implementation remains uneven and largely confined to national-level actions, reflecting the greater political feasibility of domestic measures compared to influencing decision-making processes and outputs within IEFs.

These patterns illuminate the broader progress yet persistent challenges of promoting environmental democracy at scale. Practical limits, sovereignty-based decision-making, and the absence of robust accountability mechanisms all hinder the diffusion of participatory and transparency norms across global governance architectures. Nonetheless, the evolution in the interpretation of Article 3.7 also demonstrates the adaptive potential of international environmental law to continuously strive to tackle these challenges and advance democratic values.

To realise this potential, several steps are recommended: strengthening cooperative activities under the Aarhus WGP, ensuring adequate financial and political support to the Secretariat, updating the Almaty Guidelines with targeted forum-specific guidance, and developing improved monitoring mechanisms to enhance accountability. Further case-based research could also deepen understanding of implementation practices, while examining the collective role of the European Union as a bridge between its member states and IEF participation would be especially valuable. Ultimately, while the Aarhus Convention remains regionally bounded, it can be used and further developed as a valuable testing ground and site of creative cooperation for the exportation of democratic values to the international level of environmental governance.

## Data Availability

The Aarhus Convention National Implementation Reports analysed in this study are publicly available on the Aarhus Clearinghouse for Environmental Democracy Web Portal at: https://aarhusclearinghouse.unece.org/national-reports/reports.

## Footnotes

1 Directive 2003/4/EC of 28 January 2003 on public access to environmental information [2003] OJ L41/26; Directive 2003/35/EC of 26 May 2003 on providing for public participation in respect of the drawing up of certain plans and programmes relating to the environment [2003] OJ L156/17.

2 Regulation (EU) 2021/1767 of 6 October 2021, amending Regulation (EC) No 1367/2006 on the application of the provisions of the Aarhus Convention on Access to Information, Public Participation in Decision-making and Access to Justice in Environmental Matters to Community institutions and bodies.

3 All reports available on Aarhus Clearinghouse for Environmental Democracy Web Portal at < https://aarhusclearinghouse.unece.org/national-reports/reports >. 

4 The formal deadline for submission of the latest round of NIRs was 21 May 2025. In the period between the submission deadlines for the 2021 and 2025 reporting cycles, the following changes in the treaty membership occurred: (a) Belarus withdrew on 24 October 2022; (b) Guinea-Bissau acceded on 4 April 2023; and (c) Uzbekistan acceded on 25 March 2025.

5 This survey had a low response rate from only 11 parties.

6 Albania 2025, Azerbaijan 2021, Cyprus 2021, EU 2021 and 2025, Luxembourg 2021, Portugal 2021 and 2025, Romania 2021 and 2025, Tajikistan 2021, Turkmenistan 2021, UK 2025.

7 Azerbaijan, Bosnia and Herzegovina, Finland, Italy, Moldova, Sweden.

8 Austria, Czechia, Estonia, Hungary, Kyrgyzstan.

9 Armenia, Austria, Germany, Italy.

10 Bulgaria, Denmark, Ireland, Luxembourg, Slovenia.

11 Belgium, Bulgaria, Denmark, Estonia, Finland, Ireland, Latvia, Sweden.

12 Montenegro, North Macedonia, Serbia.

13 Cyprus, Estonia, Finland, Germany, Ireland, Italy, Kazakhstan, Luxembourg, Malta, Norway, Sweden, UK.

14 Italy, North Macedonia, Bulgaria, Greece, Ukraine.

15 Bulgaria, Cyprus, Finland, Italy, Montenegro.

16 Azerbaijan, Finland, Germany, Slovenia, Sweden.

17 Belgium, Bosnia and Herzegovina, Bulgaria, Estonia, Iceland, Ireland, Italy, Luxembourg, North Macedonia, Norway, UK.

18 Austria, Georgia, Germany.
